# Prefrontal-Striatal Mechanisms of Behavioral Impulsivity During Consumption of Delayed Real Liquid Rewards

**DOI:** 10.3389/fnbeh.2021.749252

**Published:** 2021-11-08

**Authors:** Ayaka Misonou, Koji Jimura

**Affiliations:** Department of Biosciences and Informatics, Keio University, Yokohama, Japan

**Keywords:** decision-making, reward consumption, delay discounting, primary reward, human

## Abstract

Intertemporal choice involves the evaluation of future rewards and reflects behavioral impulsivity. After choosing a delayed reward in an intertemporal choice, a behavioral agent waits for, receives, and then consumes the reward. The current study focused on the consumption of the delayed reward and examined the neural mechanisms of behavioral impulsivity. In humans consuming delayed real liquid rewards in an intertemporal choice, the ventral striatum (VS) showed differential activity between anterior (aVS) and posterior (pVS) regions depending on the degree of behavioral impulsivity. Additionally, impulsive individuals showed activity in the anterior prefrontal cortex (aPFC). An analysis of task-related effective connectivity based on psychophysiological interaction (PPI) revealed that PPI was robust from the aPFC to pVS, but not in the opposite direction. On the other hand, strong bidirectional PPIs were observed between the aVS and pVS, but PPIs from the pVS to aVS were enhanced in impulsive individuals. These results suggest that behavioral impulsivity is reflected in aPFC-VS mechanisms during the consumption of delayed real liquid rewards.

## Introduction

Impulsivity is a behavioral pattern in which a behavioral agent persistently makes choices entailing a failure to achieve a long-term goal (Ainslie, 1975). In impulsive decisions, choices with optimal long-term optimal outcomes are overly discounted (Mischel et al., 1989), whereas those emphasizing short-term outcomes are overvalued (Levy and Glimcher, 2011).

One classical behavioral paradigm to evaluate impulsivity is an intertemporal choice, where a behavioral agent chooses between two alternatives that differ in outcome magnitude and time to the outcome (Rachlin et al., 1991; Keeney and Raiffa, 1993). Individuals choosing smaller rewards that are immediately available exhibit greater discounting of delayed rewards and are characterized as impulsive (Madden and Bickel, 2009). On the other hand, self-controlled (less impulsive) individuals are able to wait for a longer time showing lesser delay discounting to maximize attainment of long-term rewards (Rachlin, 2004).

The ventral striatum (VS) is implicated as a core neural site involved in impulsive decision-making (McClure et al., 2004, 2007; Tanaka et al., 2004, 2020; Jimura et al., 2013), whereas the prefrontal cortex (PFC) is associated with less impulsive (or self-controlled) decision-making (McClure et al., 2004, 2007; Shamosh et al., 2008; Hare et al., 2009; Jimura et al., 2013, 2018; Tanaka et al., 2020). These involvements of the PFC and VS raise the possibility that a PFC-VS mechanism plays an important role in determining impulsivity in value-based decision-making.

Prior human studies examining the neural mechanisms of intertemporal choice behavior have mainly focused on the choice period (McClure et al., 2004, 2007; Tanaka et al., 2004; Hariri et al., 2006; Kable and Glimcher, 2007; Peters and Buechel, 2010), with a few exceptions examining temporal changes in brain activity while future outcomes were anticipated (Berns et al., 2006; Jimura et al., 2013; McGuire and Kable, 2015; Tanaka et al., 2020). However, it remains unclear how the PFC-VS mechanism is involved while a behavioral agent is receiving a reward after having waited for it, despite the collective evidence that the VS and PFC are associated with behavioral impulsivity and self-control.

**Figure 1:**
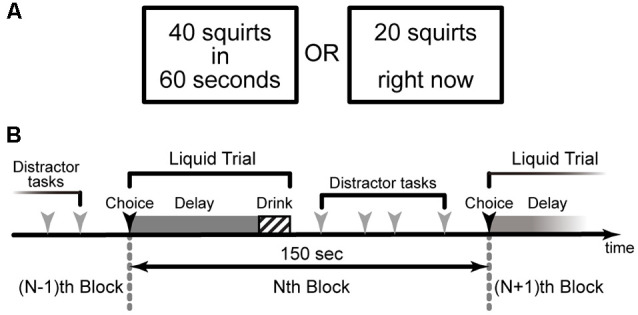
The behavioral paradigm of the intertemporal choice task for a delayed real liquid reward (Jimura et al., 2013). **(A)** Human participants made a choice between a larger amount of liquid available after a delay of 10 s of seconds and a smaller amount of liquid available immediately. **(B)** In each trial, participants consumed the real liquid rewards after experiencing a specified delay.

Intertemporal choice paradigms using delayed real liquid rewards (Jimura et al., 2009, 2011, 2013; Tanaka et al., 2020) could provide a unique opportunity to examine brain mechanisms involved in the direct experiences of delayed rewards. In these paradigms, humans make choices between two alternatives, one larger amount of liquid reward delayed by tens of seconds, and a smaller amount of liquid reward available immediately (Figure 1A). After making a choice, the participants immediately experience the delay and then consume the liquid reward (Figure 1B). Using functional MRI, we continuously measured brain activity while participants performed the paradigms (Jimura et al., 2013; Tanaka et al., 2020). Whereas our prior fMRI analyses focused on choice and delay periods (Jimura et al., 2013; Tanaka et al., 2020), in the current study, we focused on the consumption period and examined the brain mechanisms underlying impulsive choice by analyzing fMRI data while humans consumed the delayed real liquid rewards (Jimura et al., 2013). We first evaluated head movements and image quality during the drinking period, and then examined brain activity in the VS and PFC. A particular analysis focused on the anterior prefrontal cortex implicated in reward anticipation during the delay period (Jimura et al., 2013; Tanaka et al., 2020), aiming to examine prefrontal-striatum mechanisms consistently involved through entire task events in our intertemporal choice task. Finally, we assessed task-related functional connectivity between the VS and PFC.

## Materials and Methods

### Participants

Participants (*N* = 43; mean age, 23.0 years; range, 18–35 years; 20 male, 23 female) were right-handed and free from any history of psychiatric or neurological disorders. Each participant provided written informed consent after additional screening for physical or medical conditions that would affect their eligibility for fMRI. The study protocol was approved in accordance with guidelines instituted by the Washington University Human Research Protection Office, and data were collected by the senior author (KJ) at Washington University in St. Louis. Participants were compensated for their participation ($10 per h for the behavioral session, $25 per h for the fMRI session). Of the 45 participants recruited into the study, two were eliminated due to the small number of choices (<10) for the delayed option in the fMRI session.

### Dataset

We analyzed a data set collected in an fMRI experiment of an intertemporal choice task involving real liquid rewards where human participants directly experienced choice, delay, and consumption of the rewards (Jimura et al., 2013; Figure 1). In this experiment, participants performed the intertemporal decision-making task (Figure 1) in two separate (behavioral and fMRI) sessions.

The analyses of the choice and delay periods were published previously (Jimura et al., 2013), and were not analyzed in the current study. The current study analyzed the data while participants consumed the liquid rewards only, which were not analyzed in the previous study (Jimura et al., 2013).

### Behavioral Session Procedure

The behavioral session aimed to measure individuals’ delay discounting of real liquid rewards (Jimura et al., 2009, 2011, 2013). Prior to the behavioral session, participants were asked to choose one favorite drink that would serve as the reward from a list consisting of apple, orange, grape, grapefruit, and cranberry juices, lemonade, and water. No participants requested to change the reward drink in the fMRI session.

At the beginning of each trial, two alternatives were presented on the left and right sides of the screen, respectively: one involved a larger reward (20 or 40 squirts) available after a delay (10, 30, or 60 s), while the other consisted of a variable smaller amount available immediately (Figure 1A). Participants were instructed to press one of two corresponding response buttons to indicate their preference.

During the delay, a fixation cross was presented on the center of the screen. At the time of reward delivery, participants saw a visual message indicating the reward was ready. Importantly, participants were able to control the rate of liquid flow. Reward delivery continued as long as the button was held down; if the button was released, delivery paused and then resumed when the button was pressed again. During reward delivery, the amount remaining (in squirts) was displayed below a red horizontal bar whose length corresponded to the number of squirts still available. After the participant finished drinking, a fixation cross was presented.

To estimate individuals’ delay-discounting rates, the current study used three delay conditions (10, 30, 60 s) for the larger amount (40 squirts), and two delay conditions (10, 30 s) for the smaller amount (20 squirts; Jimura et al., 2009). On the first trial of each delay condition, the choice was between a larger delayed amount and an immediate reward that was half of the delayed amount. For each delay condition, the amount of the immediate reward after the first trial was adjusted based on the participant’s preceding choice. If the participant had chosen the smaller, immediate reward on the preceding trial, then the amount of the immediate reward was decreased by half (i.e., 10 and five squirts for the 40- and 20-squirt conditions, respectively); if the participant had chosen the larger, delayed reward on the preceding trial, then the amount of the immediate reward was increased by half (Jimura et al., 2009, 2011). The adjustment amount was five squirts in the third trial in the 40-squirt condition. The subjective value of the delayed reward was estimated to be 1 ml (i.e., 2.5 squirts) more or less than the amount of the immediate reward available in the last trial (third and second trials in the 40- and 20-squirt conditions, respectively), depending on whether the delayed or immediate reward had been chosen on that trial.

After the behavioral session, the participants practiced drinking liquid rewards in a supine position with a mock scanner setup. When drinking liquid rewards, they were encouraged to use jaw movements and mouth muscles for swallowing, but not to move their heads.

### fMRI Session Procedure

During fMRI scanning, participants performed an intertemporal decision-making task that was similar to that of the behavioral session. The primary difference was that the choice options for each trial were prespecified (rather than adjusted across the session), but set in an individualized manner based on a discounting profile estimated from the behavioral session. Three conditions (60 s/40 squirts, 30 s/40 squirts, 30 s/20 squirts) were used to measure brain activity during the delay period. The value of the immediate reward was systematically manipulated so that across trials, its value was smaller than the subjective value of the delayed reward, estimated for each participant based on their choice profile in the behavioral session. This manipulation of the immediate reward amount biased decisions toward delayed options, as the reward value was always smaller than the subjective value of the delayed reward, providing more opportunity to measure brain activity during consumption of delayed rewards (Jimura et al., 2013). When drinking the liquid rewards, the participants were instructed to use jaw movements and mouth muscles without moving their heads.

### Imaging Procedure

Both anatomical and functional images were available from each participant. High-resolution anatomical images were acquired using an MP-RAGE T1-weighted sequence [repetition time (TR), 9.7 s; echo time (TE), 4.0 ms; flip angle (FA), 10°; slice thickness, 1 mm; in-plane resolution, 1 × 1 mm^2^]. Functional [blood oxygen level-dependent (BOLD)] images were acquired using a gradient echo-planar imaging sequence (TR, 2.0 s; TE, 27 ms; FA, 90°; slice thickness, 4 mm; in-plane resolution, 4 × 4 mm^2^; 34 slices) in parallel to the anterior–posterior commissure line, allowing complete brain coverage at a high signal-to-noise ratio. Each functional run involved 512 volume acquisitions.

### Assessment of Impulsivity

For each participant, the degree of behavioral impulsivity was quantified by calculating the area under the discounting curve (AuC; Myerson et al., 2001; Sellitto et al., 2010; Jimura et al., 2011, 2013; Tanaka et al., 2020). The AuC represents the area under the observed subjective values at a given delay; more specifically, the AuC was calculated as the sum of the trapezoidal areas under the indifference points normalized by the amount and delay (Myerson et al., 2001). Both subjective value and delay were normalized for the purposes of calculating the AuC, which, as a result, ranged between 0.0 (maximally steep discounting) and 1.0 (no discounting). It has been argued that the AuC is the best measure of delay discounting for use in individual difference analyses, because it is theoretically neutral (i.e., assumption-free) and also psychometrically reliable (Myerson et al., 2001).

Each participant was classified into one of three groups, namely steep (STP), shallow (SHL), and intermediate (INT) discounting, based on their AuC values. The groups were identical to those analyzed in the previous study (Jimura et al., 2013).

### Image Analysis Procedure

#### Image Preprocessing

Imaging data were analyzed using SPM12^1^. All functional images were first temporally aligned across the brain volume, corrected for movement using rigid-body rotation and translation correction, and then registered to the participant’s anatomical images to correct for movement between the anatomical and function scans. Participants’ anatomical images were transformed into standardized MNI atlas space. The functional images were then registered to the reference brain using the alignment parameters derived for the anatomical scans. The data were next resampled into 2-mm isotropic voxels and spatially smoothed with an 8-mm full-width at half-maximum Gaussian kernel.

#### General Linear Model

A general linear model (GLM) approach was used to separately estimate parameter values for each event occurring during the task. Consumption of liquid rewards after the delay period was encoded as an epoch that started from the press of the button to begin drinking (i.e., initiation of pump movement) until the time at which all the liquid rewards were infused into the participants’ mouths (i.e., cessation of pump movement). As we focused on the consumption of delayed rewards, consumption periods for immediate rewards after participants chose immediate options were coded separately but similarly, and not analyzed in the current study. Choice and delay periods and distractor tasks were also included in the GLM as in the previous study (Jimura et al., 2013). All events were convolved with a canonical hemodynamic response function (HRF). In order to reduce potential confounds of head movements derived from jaw movements during drinking, head motion estimation parameters were also included in the GLM as nuisance regressors.

The parameter estimates of the consumption of delayed rewards were collected from all participants and then submitted to a group-level GLM analysis treating the participants as a random effect. To examine the correlation between AuC and the parameter estimates across participants, the AuC values of individual participants were z-scored (i.e., demeaned and divided by the standard deviation), and then included in the GLM. Additionally, for each head-movement axis, the maximum value of the movement parameters was calculated along the temporal dimension, and then z-scored across participants. The maximum movement parameters for six axes were included in the GLM as nuisance regressors to minimize potential confounds derived from head motions. Thus, the group-level GLM involved eight regressors (constant, AuC, and movement values for six axes). Z-scoring AuC and movement parameters orthogonalized these parameters and the constant regressor (group-mean effect).

**Figure 2:**
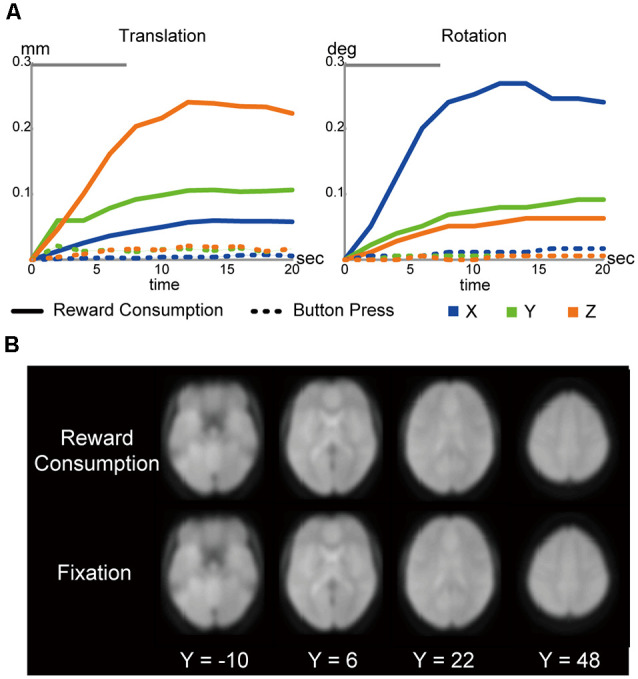
**(A)** Head motions during liquid consumptions (solid line) and button presses (dotted line; *left*: translation; *right*: rotation). The gray bars on the top indicate the mean drinking duration. **(B)** Functional images during liquid consumptions and fixation periods. The images were normalized into the MNI space, averaged across participants, and then shown in transverse sections. The levels of sections are indicated by Z levels at the bottom.

**Figure 3:**
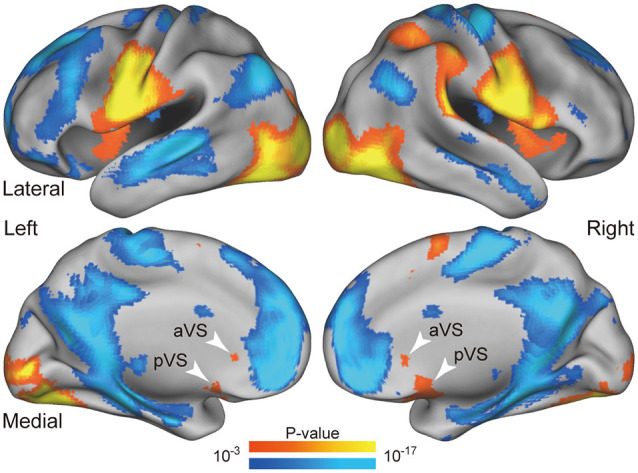
Whole-brain statistical maps for activations during liquid consumption. Statistical maps of the activations are overlaid on a 3D surface of the standard brain. The hot and cool colors indicate positive and negative activity relative to the fixation periods, respectively. The white arrowheads indicate positive activity in the anterior and posterior VS regions. aVS: anterior ventral striatum; pVS: posterior ventral striatum.

During consumption, because participants were able to press and release the button to regulate liquid flow, imaging data could be confounded by the repetitive button presses. However, as shown in Figure 2, head motion during button press is almost absent. We thus believe that button-press-derived head motions are not major confounds. Another possible confound is BOLD signal reflecting the motor execution. Importantly, as noted above, participants received a practice session after the behavioral session to drink liquid rewards in a supine position using a mock scanner. No participants had difficulty drinking the rewards. The practices enabled the participants to drink the reward without pausing liquid flows, and thus, repetitive button presses were almost absent during the drinking period. This entails that the regressor coding the button presses become almost linear to the drink-period regressor. Thus, simultaneous event coding of button press and drinking would produce significant multicollinearity. Then, we only coded drinking events in our GLM analysis to avoid statistical artifacts. We also acknowledge that the activation maps in Figure 3 involved finger movements.

#### Definitions of Regions of Interest

Because the current study focused on the mechanisms in the VS and anterior prefrontal cortex (aPFC; see also “Introduction” and “Results” sections), a region-of-interest (ROI) approach was used. ROIs were defined independently of the activation data during liquid consumption that were analyzed in the current study.

The VS ROIs were defined anatomically as spheres with 8-mm radius, centered at the bilateral anterior and posterior ends of the VS in the Harvard-Oxford MNI atlas; the spheres were further masked by the anatomical VS regions. We defined the VS ROIs for anterior and posterior parts separately, given strong activation with distinct peaks during consumption of liquid rewards, as shown in Figure 3. The anterior and posterior ROIs were labeled as the anterior VS (aVS) and posterior VS (pVS), respectively.

aPFC ROIs were defined as spheres with an 8-mm radius that were centered at bilateral aPFC coordinates showing an anticipatory utility effect during the delay period, as reported in our previous study analyzing the identical data set [coordinates: (28, 54, −7), (−31, 55, −7); Table 1 in Jimura et al., 2013]. These bilateral aPFC regions also showed the anticipatory utility effect in our recent study (Tanaka et al., 2020). For exploratory analysis, a small-volume correction approach was used, and statistical significance levels of the peak were corrected for multiple comparisons within the aPFC ROIs using voxel-level family-wise error rates.

#### Psychophysiological Interaction (PPI) Analysis

A set of PPI analyses (Friston et al., 1997) was performed to examine task-related functional connectivity among aPFC and VS regions. The seed regions for the aPFC, aVS, and pVS in each hemisphere (i.e., six ROIs in total) were identical to the ROIs defined above.

For each of the six ROIs, PPI effects were first calculated as implemented in SPM12. Then, single-level statistical analysis was performed based on a standard GLM analysis for each ROI. The GLM models included the PPI and nuisance effects (i.e., the timecourse of MRI signals in the ROI, the main effect of the condition of interest convolved with a canonical HRF, head-movement parameters for the axes, and timecourse of MRI signals for white matter, cerebrospinal fluid, and the whole-brain).

For each seed ROI, the estimated PPIs were extracted for all target ROIs (i.e., five ROIs). Thus, 30 PPIs (six seeds × five targets) were calculated for each participant. Then, these PPIs were collected from all participants, and group-level statistics were calculated for PPIs from each pair of seed and target ROIs. For statistical testing, PPIs between seed and target regions were averaged across contralateral and ipsilateral hemispheres, as we did not observe strong hemispheric asymmetry in PPIs (see “Results” section). Then, the significance of the PPI strength was tested by the one-sample *t*-test. P-values were corrected for multiple comparisons based on Bonferroni correction.

## Results

### Behavioral Results

Participants performed 19.5 ± 2.6 (mean ± SD) trials, and choose the delayed alternative in 83.0 ± 12.6% of the trials. The number of trials where the delayed alternative was chosen did not correlate with behavioral impulsivity (AuC) measured in the behavioral session (see “Materials and Methods” section) [*r* = 0.15, *t*_(41)_ = 0.97, *P* = 0.33]. The mean drinking duration was 7.5 ± 2.1 s.

Participants were classified into three groups based on AuC values reflecting the delay-discounting pattern (see “Materials and Methods”section), as in the previous study (Jimura et al., 2013: steep discounters (*N* = 15; high impulsivity), shallow discounters (*N* = 15; low impulsivity) and an intermediate group (*N* = 13).

### Head Movements During Drinking

The liquid was delivered from outside the scanner room through a plastic tube, which enabled participants to consume the liquid during fMRI administration (see “Materials and Methods” section). However, it is well known that head movements during fMRI lead to significant artifacts and signal losses in images. To evaluate whether our data were contaminated by the motion-derived artifacts and signal losses, we first assessed head movements and MRI images while participants were drinking liquid rewards.

As shown in Figure 2A, head movements were greater during liquid consumptions than during button presses made in a money discounting distractor task performed in the same scanning sessions (Figure 1B; Jimura et al., 2013, 2018). However, the absolute magnitude of head motion during consumption was small, with maximum translations of (0.06, 0.11, 0.24 mm) ± (0.02, 0.03, 0.09 mm; mean ± SD), and maximum rotations of (0.27, 0.09, 0.06) ± (0.09, 0.03, 0.02) degrees, along the *x-*, *y-*, and *z*-axes, respectively. Moreover, the maximum head movement parameters in the six axes (see “Materials and Methods” section) did not correlate with the AuC (|*r*|*s* < 0.28; |*t*|*s* < 1.88; *P*s > 0.07, uncorrected).

Nonetheless, it is known that jaw movements can yield significant instability in echoplanar images, as reported in nonhuman primate scanning (Keliris et al., 2007). However, the instability seemed absent in the current study, as we observed comparable image quality during liquid consumption and the fixation periods (Figure 2B).

Given these results, we felt confident in assuming that movement-derived contamination during liquid consumption was less obvious in the current study than the previous study (Keliris et al., 2007). Our recent study also showed similar results (Tanaka et al., 2020).

### Imaging Results

#### Brain Activity During Liquid Consumption

Figure 3 shows brain activity during consumption of delayed liquid rewards. Robust activations were observed in the primary gustatory cortex, primary motor cortex related to the jaw, and primary visual cortex, as the maps reflect various effects including jaw movements, swallowing, gustatory perception, and visual perception. These prominent activations validated the absence of major contaminations due to movement-derived artifacts and indicated that the data obtained during the consumption period could be used in substantive analyses. Importantly, robust activations were observed in the anterior and posterior parts of the bilateral VS (white arrows in Figure 3).

#### Ventral Striatal Activity and Impulsivity

As we observed stronger activity in the anterior and posterior VS (aVS and pVS, respectively) during consumption (Figure 3), we examined the activity in the aVS and pVS in each discounting group. We anatomically defined ROIs in the aVS and pVS, and evaluated consumption activity in these ROIs (see “Materials and Methods” section).

**Figure 4:**
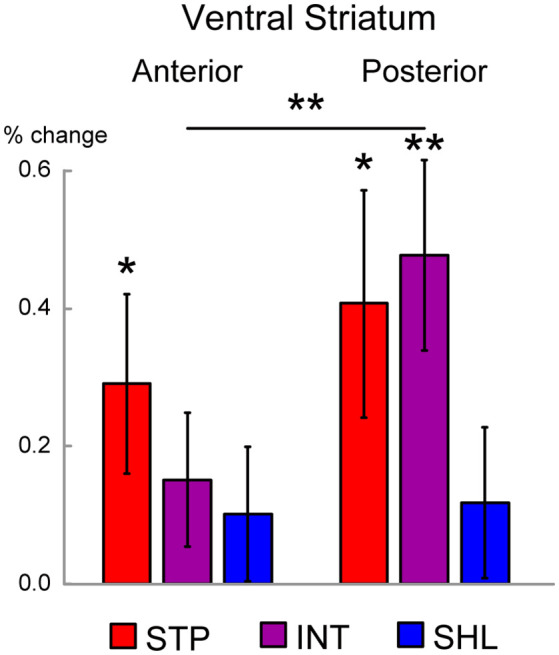
Regions of interest (ROI) analysis in the ventral striatum (VS). The magnitude of MRI signals is shown during the liquid consumption period in the anterior and posterior VS ROIs were defined anatomically. Signal magnitudes were calculated for each of the discounting groups (STP: steep; INT: intermediate; SHL: shallow), and averaged across hemispheres. Error bars indicate SEM. Levels of statistical significance are indicated by the number of asterisks (**P < 0.01; *P < 0.05).

As shown in Figure 4, steep discounters (high impulsivity) showed significant activation in the aVS, [*t*_(14)_ = 2.2; *P* < 0.05]. On the other hand, both steep and intermediate discounters showed significant activation in the pVS [steep: *t*_(14)_ = 2.5; *P* < 0.05; intermediate: *t*_(12)_ = 3.5; *P* < 0.01]. Interestingly, in the intermediate discounters, the activation was greater in the pVS than the aVS [*t*_(12)_ = 3.1; *P* < 0.01]. On the other hand, in both the aVS and pVS, significant activation was absent in shallow discounters (low impulsivity). These results suggest that the aVS and pVS are differently involved in liquid reward consumption depending on the degree of impulsivity.

#### Prefrontal Activity and Impulsivity

Aiming to examine neural mechanisms consistently involved through task events, we asked how the anterior prefrontal region related to reward anticipation during the delay (Jimura et al., 2013; Tanaka et al., 2020) was involved during consumption.

**Figure 5:**
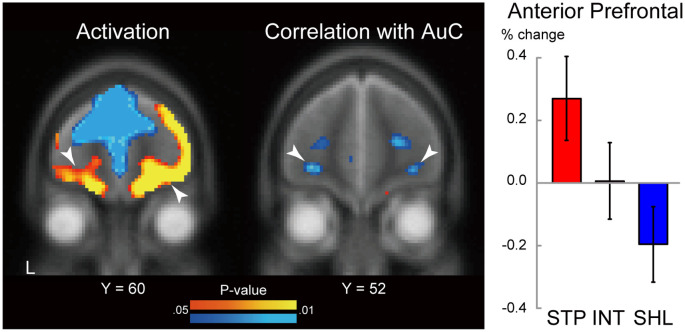
Statistical map for brain activity during liquid consumption (*left*). The level of the section is indicated by the Y coordinate of the MNI space. The threshold of the map was *P* < 0.05 (uncorrected) for display purposes. White arrow heads indicate correlations in the anterior prefrontal cortex. L: left. Hot and cool colors indicate positive and negative activation, respectively. Statistical map for correlation between behavioral impulsivity and brain activity during liquid consumption (*middle*). Behavioral impulsivity is quantified as the area under the curve of the subjective value of the delayed reward. The format is similar to those on the left. Hot and cool colors indicate positive and negative correlation, respectively. MRI signal magnitudes in the aPFC ROIs were calculated for each discounting group and averaged across hemispheres (*right*). Regions of interest were defined based on the previous study (Jimura et al., 2013). The formatting is similar to that in Figure 4.

We first explored brain regions showing significant activation during consumption within aPFC ROIs. The ROIs were defined based on our previous study (Jimura et al., 2013; see also “Materials and Methods” section). As shown in Figure 5 (*left*), aPFC regions showed strong activation bilaterally [*P* < 0.05, corrected for multiple comparison based on voxel-level family-wise error rate; left: (−32, 62, −6), *z* = 2.6; right: (26, 58, −1), *z* = 3.0].

We next examined the correlation between the consumption period activity and the degree of delay discounting estimated in a separate behavioral session (see “Materials and Methods” section). Specifically, we explored aPFC ROIs showing the correlation between AuC and brain activity during consumptions. As shown in Figure 5 (*middle*), negative correlations were observed in the bilateral aPFC [*P* < 0.05, corrected for multiple comparison based on voxel-level family-wise error rate; left: (−28, 52, −8), *z* = 2.5; right: (32, 48), −6, *z* = 2.4]. Importantly, these aPFC regions were identified within the identical ROIs involving regions showing strong activation. The negative correlations indicate greater activity in steep discounters (high impulsivity; Figure 5 *right*). In impulsive individuals, the aPFC activity was almost significant [*t*_(14)_ = 2.0, *P* = 0.065], possibly due to the small sample size of each discounting group, and strong activation was absent when averaging across all participants [*t*_(42)_ = 0.52, *P* = 0.62].

Interestingly, in the previous studies, the aPFC regions exhibited an anticipatory utility effect during the delay period and the effect was enhanced in shallow discounters (Jimura et al., 2013; Tanaka et al., 2020), whereas in the current study, the consumption period activity was reduced in shallow discounters (Figure 5 *left*; see “Discussion” section for details).

#### Psychophysiological Interaction

The results related to consumption period activity and its relation to behavioral impulsivity suggest that the aPFC and VS play an important role during the consumption of real liquid rewards. We, therefore, examined task-related functional connectivity between these regions based on psychophysiological interactions (PPIs; Friston et al., 1997; see “Materials and Methods” section).

**Figure 6:**
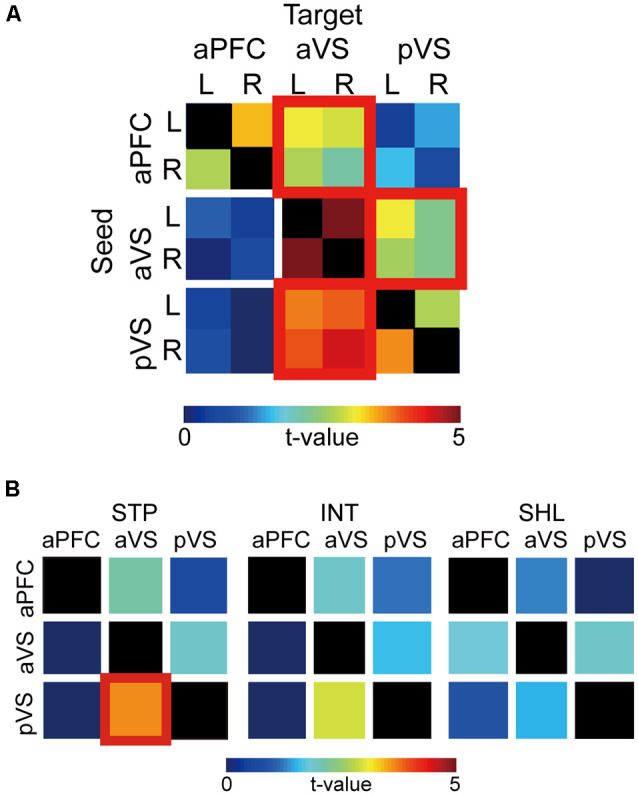
Psychophysiological interaction (PPI) analysis. **(A)** Group level *t*-values of PPIs from seed to target regions are color-coded according to the bar at the bottom. The columns and rows indicate seeds and targets, respectively. aPFC: anterior prefrontal cortex; aVS: anterior ventral striatum; pVS: posterior ventral striatum. Significant PPIs are indicated by red squares. L: left; R: right. **(B)** PPIs for each discounting group. Formatting is similar to that in panel **(A)**.

Figure 6A shows PPIs between the aPFC, aVS, and pVS. For each pair of ROIs, PPIs appear to covary between ipsilateral and contralateral hemispheres, and obvious hemispheric asymmetries look absent. Thus, PPIs were averaged across hemispheres, and statistical testing was performed. From the aPFC, PPI was strong towards aVS [*t*_(42)_ = 3.1; *P* < 0.05, Bonferroni corrected], but strong PPI was not observed in the opposite direction, suggesting top-down signaling from the aPFC to aVS. On the other hand, PPIs were robust bidirectionally between the aVS and pVS [aVS to pVS: *t*_(42)_ = 3.1; *P* < 0.05, Bonferroni corrected; pVS to aVS: *t*_(42)_ = 4.6; *P* < 0.0001, Bonferroni corrected].

In order to examine whether the PPIs were dependent on impulsivity, the bidirectional PPI matrix was inspected for the steep, intermediate, shallow discounting groups. As shown in Figure 6B, in steep discounters, there was strong PPI from the pVS to aVS [*t*_(42)_ = 3.7; *P* < 0.001, Bonferroni corrected], but not in the opposite direction. Such unidirectional strong PPI was absent in the other two groups.

## Discussion

The current study provides new insights regarding prefrontal-striatal mechanisms of intertemporal choice by focusing on brain activity and task-related functional connectivity while humans consumed real liquid rewards delayed by tens of seconds. Impulsivity was associated with activity enhancement in the aPFC and VS, and the activation magnitudes in the VS differed between the anterior and posterior regions depending on impulsivity. PPI was robust from the aPFC to aVS, but not in the opposite direction, suggesting top-down signaling from aPFC to aVS. On the other hand, bidirectional PPIs were observed between the aVS and pVS, with enhanced PPI from the pVS to aVS in impulsive individuals (steep discounters). These findings suggest that prefrontal and striatal mechanisms are involved in reward consumption, reflecting behavioral impulsivity in decision-making.

### A Putative Prefrontal-Striatal Model of Impulsivity During Reward Consumption

**Figure 7:**
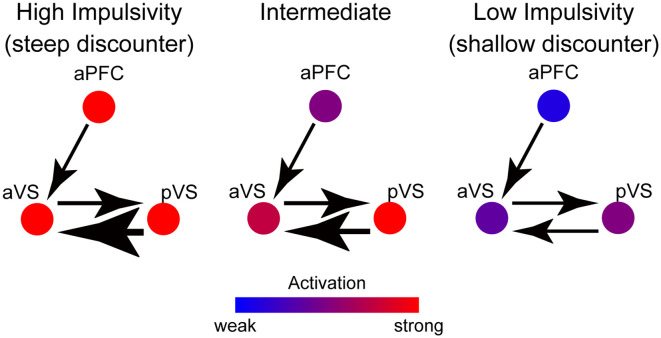
A schematic path diagram for putative functional mechanisms between the aPFC, aVS, and pVS during reward consumption. The colors of the circles indicate the activation magnitude in these regions, according to the color bar at the bottom. The thickness of each arrow indicates the strength of the connectivity from region to region, and the direction of each arrowhead indicates the signal flow direction. In highly impulsive individuals, strong aPFC and pVS signals are sent to the aVS, enhancing aVS activity, whereas, in minimally impulsive individuals, negative aPFC activation reduces aVS activity.

The current results highlight functional segregation and integration in the prefrontal cortex and the ventral striatum during the consumption of delayed liquid rewards. Figure 7 summarizes our results and illustrates activity magnitudes and signal flows between the aPFC, aVS, and pVS for three levels of impulsivity.

In the aPFC, activation is greater in impulsive individuals, resulting in enhanced signaling toward the aVS and an elevation of its activity. On the other hand, the pVS transmits reward-related signals to the aVS, and then aVS activity is further amplified in impulsive individuals. Interestingly, the signals from both the aPFC and pVS appear to be associated with both the magnitude of aVS activation and the level of impulsivity.

These mechanisms can be interpreted as showing that the reward-related signals from the aPFC and pVS are aggregated into the aVS, which is critical for the degree of behavioral impulsivity. These prefrontal-striatum mechanisms are compatible with those suggested by prior studies analyzing connectivity between aPFC and VS (Diekhof and Gruber, 2010; Jimura et al., 2013; Tanaka et al., 2020).

A diffusion tensor imaging study of the human VS showed that both the anterior and posterior parts of the VS are connected to the anterior ventral part of the PFC and the orbitofrontal cortex, including the aPFC in the current study. Interestingly, the anterior part is also connected to more dorsal parts of the PFC (Tziortzi et al., 2014). The dorsal parts of the PFC are involved in working memory and choice difficulty in intertemporal choice, which is enhanced in self-controlled individuals (Jimura et al., 2018). The anatomical connection between the anterior VS and dorsal PFC may regulate the aPFC-aVS-pVS mechanisms during consumption of liquid rewards, as illustrated by the reduced activity in the intermediate discounting group (Figure 4).

### aPFC-VS Mechanisms in Distinct Behavioral Phases

The current study focused on the consumption phase of intertemporal choice, whereas previous studies analyzed delay and choice phases (Jimura et al., 2013; Tanaka et al., 2020). These collective results are derived from a single dataset, allowing us to speculate regarding possible functional mechanisms involved in the distinct phases of intertemporal choice behavior: choice, delay, and consumption.

In all three phases, impulsivity in decision-making was associated with increased VS activation, consistent with previous reports (Tanaka et al., 2004; Hariri et al., 2006; Kable and Glimcher, 2007; Ballard and Knutson, 2009; Pine et al., 2009). On the other hand, aPFC activation differed among the phases. During the choice and early delay periods, self-controlled (less impulsive) individuals exhibited increased aPFC activity (Jimura et al., 2013; Tanaka et al., 2020). In contrast, in the current study, highly impulsive individuals exhibited increased aPFC activity during the consumption period. A possible unified explanation of these findings is that the aPFC is associated with currently available utility, i.e., future anticipation during delay and reward consumption during drinking. The former may be more valuated in self-controlled individuals to maximize future reward attainment, whereas the latter may be evaluated in impulsive individuals when consuming a reward after a delay.

Greater aPFC activation during a choice period in less impulsive individuals may reflect resistance to impulsive choice, which reduces VS activity (Diekhof and Gruber, 2010). During the delay period, aPFC regions may encode an anticipatory utility signal associated with delayed rewards, which is the extra utility derived from the pleasure of waiting for a reward delivered in the future (Loewenstein, 1987; Berns et al., 2006, 2007; Peters and Buechel, 2010; Jimura et al., 2013; Tanaka et al., 2020).

On the other hand, as mentioned above, highly impulsive individuals showed greater aPFC activation during the consumption period, suggesting marked current utility when individuals consume liquid rewards. Because highly impulsive individuals do not prefer to wait to obtain a larger reward, the utility of a liquid reward would become greater upon completion of the delay, eliciting greater aPFC activation when consuming delayed rewards.

Another possibility is that the aPFC encodes current pleasure related to reward attainment, as suggested by aPFC regions showing anticipatory utility effect that is thought to reflect pleasure of waiting (Loewenstein, 1987; Jimura et al., 2013; Tanaka et al., 2020). Thus, the receipt of a delayed reward may provide greater pleasure for highly impulsive individuals. Alternatively, while consuming the reward, highly impulsive individuals may retrieve episodic information about past experiences of rewards, resulting in greater aPFC activation. However, additional evidence is needed to directly support the role of the aPFC in relation to pleasure.

## Conclusion

The current study addressed a unique question, how the prefrontal cortex and ventral striatum are involved while humans consumed delayed real liquid rewards. We found that the anterior prefrontal cortex, anterior ventral striatum, and posterior ventral striatum constitute a functional network, which is modulated by behavioral impulsivity. Our results highlight a prefrontal-striatal mechanism of behavioral impulsivity during reward consumption.

## Data Availability Statement

The datasets are available from the corresponding author on reasonable request. Requests to access these datasets should be directed to Koji Jimura, koji.jimura@gmail.com.

## Ethics Statement

The studies involving human participants were reviewed and approved by Washington University in St. Louis, USA. Patients/participants provided their written informed consent to participate in this study.

## Author Contributions

KJ designed research. AM and KJ performed research, analyzed data, wrote the article. All authors contributed to the article and approved the submitted version.

## Conflict of Interest

The authors declare that the research was conducted in the absence of any commercial or financial relationships that could be construed as a potential conflict of interest.

## Publisher’s Note

All claims expressed in this article are solely those of the authors and do not necessarily represent those of their affiliated organizations, or those of the publisher, the editors and the reviewers. Any product that may be evaluated in this article, or claim that may be made by its manufacturer, is not guaranteed or endorsed by the publisher.

## Footnotes

^1^ https://www.fil.ion.ucl.ac.uk/spm/

## References

[B1] Ainslie G. (1975). Specious reward - behavioral theory of impulsiveness and impulse control. Psychol. Bull. 82, 463–496. 10.1037/h0076860 1099599

[B2] Ballard K. Knutson B. (2009). Dissociable neural representations of future reward magnitude and delay during temporal discounting. Neuroimage 45, 143–150. 10.1016/j.neuroimage.2008.11.004 19071223 PMC2685201

[B3] Berns G. S. Chappelow J. Cekic M. Zink C. F. Pagnoni G. Martin-Skurski M. E. (2006). Neurobiological substrates of dread. Science 312, 754–758. 10.1126/science.1123721 16675703 PMC1820741

[B4] Berns G. S. Laibson D. Loewenstein G. (2007). Intertemporal choice - toward an integrative framework. Trends. Cogn. Sci. 11, 482–488. 10.1016/j.tics.2007.08.011 17980645

[B6] Diekhof E. K. Gruber O. (2010). When desire collides with reason: functional interactions between anteroventral prefrontal cortex and nucleus accumbens underlie the human ability to resist impulsive desires. J. Neurosci. 30, 1488–1493. 10.1523/JNEUROSCI.4690-09.2010 20107076 PMC6633806

[B7] Friston K. J. Buechel C. Fink G. R. Morris J. Rolls E. Dolan R. J. (1997). Psychophysiological and modulatory interactions in neuroimaging. Neuroimage 6, 218–229. 10.1006/nimg.1997.0291 9344826

[B8] Hare T. A. Camerer C. F. Rangel A. (2009). Self-control in decision-making involves modulation of the vmPFC valuation system. Science 324, 646–648. 10.1126/science.1168450 19407204

[B9] Hariri A. R. Brown S. M. Williamson D. E. Flory J. D. de Wit H. Manuck S. B. (2006). Preference for immediate over delayed rewards is associated with magnitude of ventral striatal activity. J. Neurosci. 26, 13213–13217. 10.1523/JNEUROSCI.3446-06.2006 17182771 PMC6675016

[B10] Jimura K. Chushak M. S. Braver T. S. (2013). Impulsivity and self-control during intertemporal decision making linked to the neural dynamics of reward value representation. J. Neurosci. 33, 344–357. 10.1523/JNEUROSCI.0919-12.2013 23283347 PMC3711626

[B11] Jimura K. Chushak M. S. Westbrook A. Braver T. S. (2018). Intertemporal decision-making involves prefrontal control mechanisms associated with working memory. Cereb Cortex 28, 1105–1116. 10.1093/cercor/bhx015 28174915 PMC6454524

[B12] Jimura K. Myerson J. Hilgard J. Braver T. S. Green L. (2009). Are people really more patient than other animals? Evidence from human discounting of real liquid rewards. Psychon. Bull. Rev. 16, 1071–1075. 10.3758/PBR.16.6.1071 19966257 PMC3886190

[B13] Jimura K. Myerson J. Hilgard J. Keighley J. Braver T. S. Green L. (2011). Domain independence and stability in young and older adults’ discounting of delayed rewards. Behav. Proc. 87, 253–259. 10.1016/j.beproc.2011.04.006 21550384 PMC3138910

[B14] Kable J. W. Glimcher P. W. (2007). The neural correlates of subjective value during intertemporal choice. Nat. Neurosci. 10, 1625–1633. 10.1038/nn2007 17982449 PMC2845395

[B15] Keeney R. L. Raiffa H. (1993). Decisions with Multiple Objectives: Preferences and Value Tradeoffs. New York: Wiley.

[B16] Keliris G. A. Shmuel A. Ku S.-P. Pfeuffer J. Oeltermann A. Steudel T.. (2007). Robust controlled functional MRI in alert monkeys at high magnetic field: effects of jaw and body movements. Neuroimage 36, 550–570. 10.1016/j.neuroimage.2007.02.057 17509896

[B17] Levy D. J. Glimcher P. W. (2011). Comparing apples and oranges: using reward-specific and reward-general subjective value representation in the brain. J. Neurosci. 31, 14693–14707. 10.1523/JNEUROSCI.2218-11.2011 21994386 PMC3763520

[B18] Loewenstein G. (1987). Anticipation and the valuation of delayed consumption. Econ. J. 97, 666–684. 10.2307/2232929

[B19] Madden G. J. Bickel W. K. (2009). Impulsivity: The Behavioral and Neurological Science of Discounting. Washington, DC: American Psychological Association.

[B20] McClure S. M. Ericson K. M. Laibson D. I. Loewenstein G. Cohen J. D. (2007). Time discounting for primary rewards. J. Neurosci. 27, 5796–5804. 10.1523/JNEUROSCI.4246-06.2007 17522323 PMC6672764

[B21] McClure S. M. Laibson D. I. Loewenstein G. Cohen J. D. (2004). Separate neural systems value immediate and delayed monetary rewards. Science 306, 503–507. 10.1126/science.1100907 15486304

[B22] McGuire J. T. Kable J. W. (2015). Medial prefrontal cortical activity reflects dynamic re-evaluation during voluntary persistence. Nat. Neurosci. 18, 760–766. 10.1038/nn.3994 25849988 PMC4437670

[B23] Mischel W. Shoda Y. Rodriguez M. L. (1989). Delay of gratification in children. Science 244, 933–938. 10.1126/science.2658056 2658056

[B24] Myerson J. Green L. Warusawitharana M. (2001). Area under the curve as a measure of discounting. J. Exp. Anal. Behav. 76, 235–243. 10.1901/jeab.2001.76-235 11599641 PMC1284836

[B25] Peters J. Buechel C. (2010). Episodic future thinking reduces reward delay discounting through an enhancement of prefrontal-mediotemporal interactions. Neuron 66, 138–148. 10.1016/j.neuron.2010.03.026 20399735

[B26] Pine A. Seymour B. Roiser J. P. Bossaerts P. Friston K. J. Curran H. V.. (2009). Encoding of marginal utility across time in the human brain. J. Neurosci. 29, 9575–9581. 10.1523/JNEUROSCI.1126-09.2009 19641120 PMC2816907

[B27] Rachlin H. (2004). The Science of Self-control. Cambridge: Harvard University Press.

[B28] Rachlin H. Raineri A. Cross D. (1991). Subjective-probability and delay. J. Exp. Anal. Behav. 55, 233–244. 10.1901/jeab.1991.55-233 2037827 PMC1323057

[B29] Sellitto M. Ciaramelli E. di Pellegrino G. (2010). Myopic discounting of future rewards after medial orbitofrontal damage in humans. J. Neurosci. 30, 16429–16436. 10.1523/JNEUROSCI.2516-10.2010 21147982 PMC6634874

[B30] Shamosh N. A. DeYoung C. G. Green A. E. Reis D. L. Johnson M. R. Conway A. R. A.. (2008). Individual differences in delay discounting relation to intelligence, working memory and anterior prefrontal cortex. Psychol. Sci. 19, 904–911. 10.1111/j.1467-9280.2008.02175.x 18947356

[B32] Tanaka D. Aoki R. Suzuki S. Takeda M. Nakahara K. Jimura K. (2020). Self-controlled choice arises from dynamics prefrontal signals that enable future anticipation. J. Neurosci. 40, 9736–9750. 10.1523/JNEUROSCI.1702-20.2020 33188069 PMC7726527

[B31] Tanaka S. C. Doya K. Okada G. Ueda K. Okamoto Y. Yamawaki S. (2004). Prediction of immediate and future rewards differentially recruits cortico-basal ganglia loops. Nat. Neurosci. 7, 887–893. 10.1038/nn1279 15235607

[B33] Tziortzi A. C. Haber S. N. Searle G. E. Tsoumpas C. Long C. J. Shotbolt P.. (2014). Connectivity-based functional analysis of dopamine release in the striatum using diffusion-weighted MRI and positron emission tomography. Cereb. Cortex 24, 1165–1177. 10.1093/cercor/bhs397 23283687 PMC3977617

